# Utilizing longitudinal microbiome taxonomic profiles to predict food allergy via Long Short-Term Memory networks

**DOI:** 10.1371/journal.pcbi.1006693

**Published:** 2019-02-04

**Authors:** Ahmed A. Metwally, Philip S. Yu, Derek Reiman, Yang Dai, Patricia W. Finn, David L. Perkins

**Affiliations:** 1 Department of Bioengineering, University of Illinois at Chicago, Chicago, Illinois, United States of America; 2 Department of Computer Science, University of Illinois at Chicago, Chicago, Illinois, United States of America; 3 Department of Medicine, University of Illinois at Chicago, Chicago, Illinois, United States of America; 4 Department of Microbiology/Immunology, University of Illinois at Chicago, Chicago, Illinois, United States of America; 5 Department of Surgery, University of Illinois at Chicago, Chicago, Illinois, United States of America; Rutgers University, UNITED STATES

## Abstract

Food allergy is usually difficult to diagnose in early life, and the inability to diagnose patients with atopic diseases at an early age may lead to severe complications. Numerous studies have suggested an association between the infant gut microbiome and development of allergy. In this work, we investigated the capacity of Long Short-Term Memory (LSTM) networks to predict food allergies in early life (0-3 years) from subjects’ longitudinal gut microbiome profiles. Using the DIABIMMUNE dataset, we show an increase in predictive power using our model compared to Hidden Markov Model, Multi-Layer Perceptron Neural Network, Support Vector Machine, Random Forest, and LASSO regression. We further evaluated whether the training of LSTM networks benefits from reduced representations of microbial features. We considered sparse autoencoder for extraction of potential latent representations in addition to standard feature selection procedures based on Minimum Redundancy Maximum Relevance (mRMR) and variance prior to the training of LSTM networks. The comprehensive evaluation reveals that LSTM networks with the mRMR selected features achieve significantly better performance compared to the other tested machine learning models.

## Author summary

Deep learning has become a powerful methodology to derive knowledge from the vast amount of data available from biomedical studies. However, successful applications of deep learning models to clinical studies remain challenging due to the complexity of biological data generated from the new experimental platforms as well as the inconsistency in clinical sample collection in a longitudinal study. The present work introduces our results using Long Short-Term Memory (LSTM) networks for the prediction of infant food allergy based on gut microbiome data collected from infants and young children. Our framework is empowered by the LSTM’s capacity for learning the dependency on gut microbiome longitudinal observations. The LSTM performance can be further improved by incorporating procedures of feature selection such as Minimum Redundancy Maximum Relevance. Promising performance of our new approach is demonstrated through the comparison with several traditional machine learning models. Our study suggests that LSTM network learning may be useful to other longitudinal microbiome data for prediction of a subject’s clinical outcome.

This is a *PLoS Computational Biology* Methods paper.

## Introduction

Food sensitization and allergy are characterized by an immunologic reaction caused by exposure to antigenic products derived from food, such as Ara h1 (peanuts) or tropomyosin (shellfish). The estimated prevalence of food sensitization and allergy in the US is 8% [1], with peak prevalence between the ages of one and two years old. Food sensitization is often associated with a positive reaction to skin prick testing or by increased levels of serum specific Immunoglobulin E (IgE) to specific food antigens. Food allergy can be diagnosed by the clinical history of symptoms after food ingestion or by direct food challenge and monitoring of symptoms. Notably, not all individuals who are sensitized develop allergy, but the prevalence of food allergy is substantially higher for individuals with food sensitization. In turn, not all individuals with food allergy are sensitized to food allergens and thus serologic or skin testing alone is not sufficient for diagnosis of the food allergy. There is a need for more objective measures that have predictive value in diagnosing food allergy.

Food allergies are categorized into three groups: IgE-mediated, non-IgE-mediated, and mixed reactions. IgE-mediated food reactions are caused by the cross-linking of IgE on the surface of mast cells or basophils by food proteins. This leads to rapid degranulation of these cells and release of histamine which is the primary mediator of IgE symptoms including urticaria, angioedema, and anaphylaxis, which can be life-threatening. These symptoms present acutely within minutes after the ingestion of food allergen. In contrast, non-IgE-mediated and mixed reactions present in a subacute to chronic time-frame and their mechanisms are less defined. Subacute symptoms associated with non-IgE food allergy are localized to the gastrointestinal tract, such as blood/mucus filled stools or vomiting, which can lead to chronic symptoms such as weight loss, dehydration, lethargy, and failure to thrive. Mixed reactions are characterized by food allergens exacerbating IgE-mediated diseases, such as atopic dermatitis. Thus, food allergy represents a spectrum of diseases that are currently diagnosed by subjective measurements during early life.

The increasing incidence of food allergy and other allergic diseases has been attributed to “westernized” lifestyles, as prevalence of these diseases is substantially higher in the developed world. One over-arching theme as to why the incidence of allergy is increasing is the loss or disturbance in communities of micro-organisms that live on and in us (i.e., the microbiome). Importantly, differences in composition of the gut microbiome have been associated with food sensitization and/or IgE and non-IgE-mediated reactions [2–7], symptom resolution [8], and prevention and treatment [9, 10]. This opens the door to develop more rigorous food allergy prediction models that are based on gut microbiome profiles of newborns, which could be used to predict food allergy and inform early intervention with novel therapies.

Longitudinal microbiome studies have been widely utilized to study disease prognosis and microbial dynamics within an ecosystem such as the gut, lung, or kidney [11–16]. The exponential reduction in sequencing cost has resulted in the increase in popularity of longitudinal microbiome studies. Usually, a microbiome study is performed by sequencing the extracted DNA from a biological sample using either metagenomic shotgun (MGS) or 16S rRNA gene sequencing [17]. Metagenomic reads are processed for each sample independently to construct the taxonomic and/or functional profiles [18–20]. Developing methods that predict the host phenotype from longitudinal microbiome samples comes with some challenges, e.g., variable sample collection times and an uneven number of timepoints along the subjects’ longitudinal time-line, especially when samples are collected from human subjects [21]. Hence, using standard prediction methods such as Hidden Markov Models (HMMs) [22] and Auto Regressive (AR) models [23] may not be suitable in these cases.

Deep learning has revolutionized various fields by offering robust strategies to extract abstract nonlinear features that are refractory to traditional methods [24, 25]. Multiple deep learning frameworks have been developed to predict phenotype from snapshot microbiome profiles [26–29]. On the other hand, a powerful approach to analyze temporal data is the Recurrent Neural Network (RNN). RNNs have shown success in different fields such as natural language processing [30] and speech recognition [31]. Although in theory, the RNN can learn dependent representation from distant events, it fails in practice due to problems with vanishing gradients [25]. This problem occurs because the error loss is back-propagated through the deep network by multiplying the derivative of the utilized activation function, which is usually the sigmoid or hyperbolic tangent. The derivative of these activation functions is usually less than one. Hence, multiplying the error loss by many of these less than one numbers causes the vanishing gradient problem. Fortunately, Long Short-Term Memory (LSTM) networks, a modified variant of the RNN, have the ability to learn temporal behavior for a time series event and to overcome the vanishing gradient problem in standard RNNs [32].

In this work, we present a deep learning framework to predict food allergy in infants based on longitudinal gut microbiome profiles. In our model, we use all historical samples up to timepoint *t* (features at timepoint *t* included) from each subject to predict the phenotype (food allergy vs. non-food allergy) at timepoint *t*. We hypothesize that adding the information from past gut microbiome profiles increases the predictive power of food allergy versus training a model with each timepoint independently. The proposed framework is based on LSTM networks and is flexible such that it can analyze subjects with a different number of timepoints.

## Materials and methods

**Fig 1:**
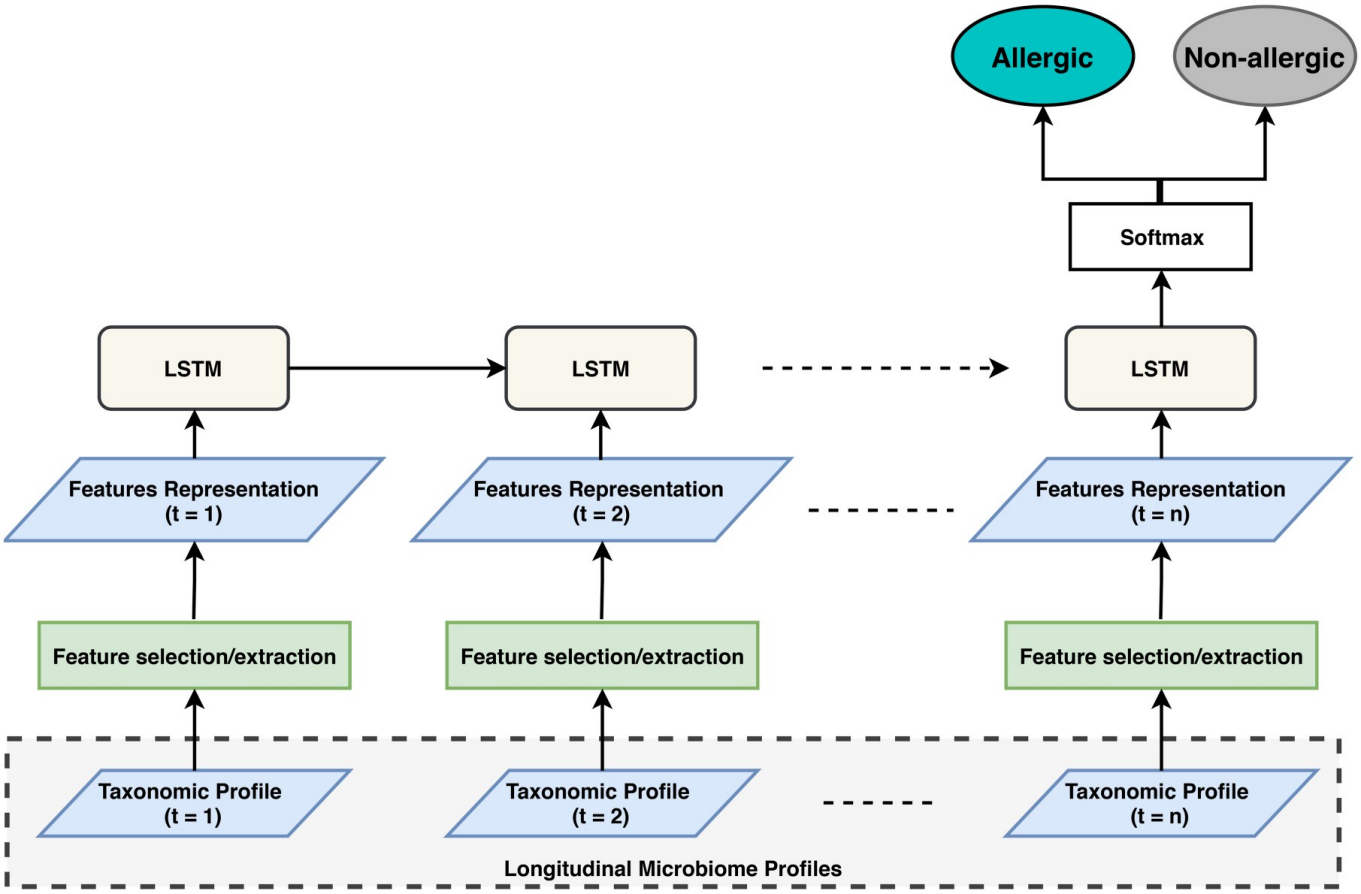
The proposed deep learning framework, where *n* denotes the number of timepoints from each subject, which is not the same for all subjects.

Fig 1 illustrates an overview of our proposed framework to predict food allergy from longitudinal gut microbiome taxonomic profiles. It consists of two main modules; a feature selection/extraction module and an LSTM network. The input to the feature selection/extraction module is a vector representing a normalized taxonomic profile of a subject’s microbial sample. The selected or extracted features are then passed to the LSTM module to learn temporal dependency between sequence profiles. Subsequently, the output from the last cell of the LSTM model is then fed to a softmax output layer where the prediction can be determined (e.g., food allergy vs. non-food allergy). The methodology of learning temporal dependency and obtaining the features’ representation is explained in details in the following sections.

### Learning temporal context via Long Short-Term Memory (LSTM) networks

The LSTM network is a variant of the vanilla RNN that has the ability to learn long sequences [32]. This ability is due to the presence of a memory, usually referred to as Cell state (*C*), that stores long-term information so that errors will not be propagated through distant states. LSTM networks solve the two major problems of RNNs, the vanishing and exploding gradient descent problems. It accomplishes this by using 3 gates to control the cell state: forget, input, and output gates. The forget gate (**f**_*t*_) controls the amount of information that should be forgotten from the previous cell state by analyzing the current input **x**_*t*_ and the previously hidden state **h**_*t*−1_. The input gate (**i**_*t*_) controls how much of the current input **x**_*t*_ should be used in training. Then, a list of new candidates ($\tilde{C}$) for the cell state is calculated as in Eq 1, where **W**_*c*_ and **U**_*c*_ are weight matrices, and **b**_*c*_ is a bias term. Updating the cell state is performed as formulated in Eq 2.

$$\begin{array}{c} \tilde{C}=\tanh\left(W_{c}x_{t}+U_{c}h_{t-1}+b_{c}\right) \end{array}$$ (1)

$$\begin{array}{c} C_{t}=f_{t}C_{t-1}+i_{t}\tilde{C} \end{array}$$ (2)

To calculate the hidden state (**h**_*t*_) of an LSTM unit that is passed to the next sample in a sequence, the output of the output gate (**o**_*t*_, Eq 3) is multiplied by the squashed cell state **C**_*t*_ via tanh function (Eq 4), where **W**_*o*_ and **U**_*o*_ are weight matrices, **b**_*o*_ is a bias term, and $\sigma \left(x\right)=\frac{1}{1+e^{-x}}$.

$$\begin{array}{c} o_{t}=\sigma \left(W_{o}x_{t}+U_{o}h_{t-1}+b_{o}\right) \end{array}$$ (3)

$$\begin{array}{c} h_{t}=o_{t}*\tanh\left(C_{t}\right) \end{array}$$ (4)

Since the number of samples for each subject is not identical, we extract the LSTM output of the last sample of each subjects’ sequence. This output is then fed into a dense layer with a linear activation function and with the dimension of the number of hidden neurons by the number of classes (64x2 in our case). The output of the dense layer (**z**_*t*_) is then fed to a softmax function in order to give an output probability for each class (${\hat{y}}_{t}=\text{softmax}\left(z_{t}\right)$). The class with higher probability is considered the predicted class. Because our main target in this study is to predict the phenotype (food allergic vs. non-food allergic), we used the cross-entropy ($E(y_{t_{i}},{\hat{y}}_{t_{i}})$) between target (**y**_*t*_) and predicted output (${\hat{y}}_{t}$) in the loss function (Eq 5), where *N* denotes the number of data sequences (subjects in our case). To prevent overfitting, we used L2 regularization in the loss function (Eq 5), where *J* = {*f*, *i*, *c*, *o*, *z*}. We used the back-propagation algorithm to minimize the loss function (*Loss*^(1)^).

$${\text{Loss}}^{\left(1\right)}\left(y_{t},{\hat{y}}_{t}\right)=\sum_{i=1}^{N}\text{E}\left(y_{t_{i}},{\hat{y}}_{t_{i}}\right)+\lambda \sum_{j\in J}^{}{\left\|W_{j}\right\|}^{2}$$ (5)

### Feature selection or extraction procedures

Since microbial profiles usually consist of hundreds or thousands of features, it is of importance to select or extract the most meaningful features to increase the model predictive power. In this study, we investigated the incorporation of sparse autoencoder to learn a compressed latent representation of a sample’s microbial features. Besides sparse autoencoders, we tried various traditional feature selection methods, such as Minimum Redundancy Maximum Relevance (mRMR) [33], and selecting the top variable features. A major advantage of mRMR and the selection of top variable features over autoencoders is the interpretability of the selected features. On the other hand, autoencoders have the advantage of extracting compressed latent representation of high dimensional datasets.

**Fig 2:**
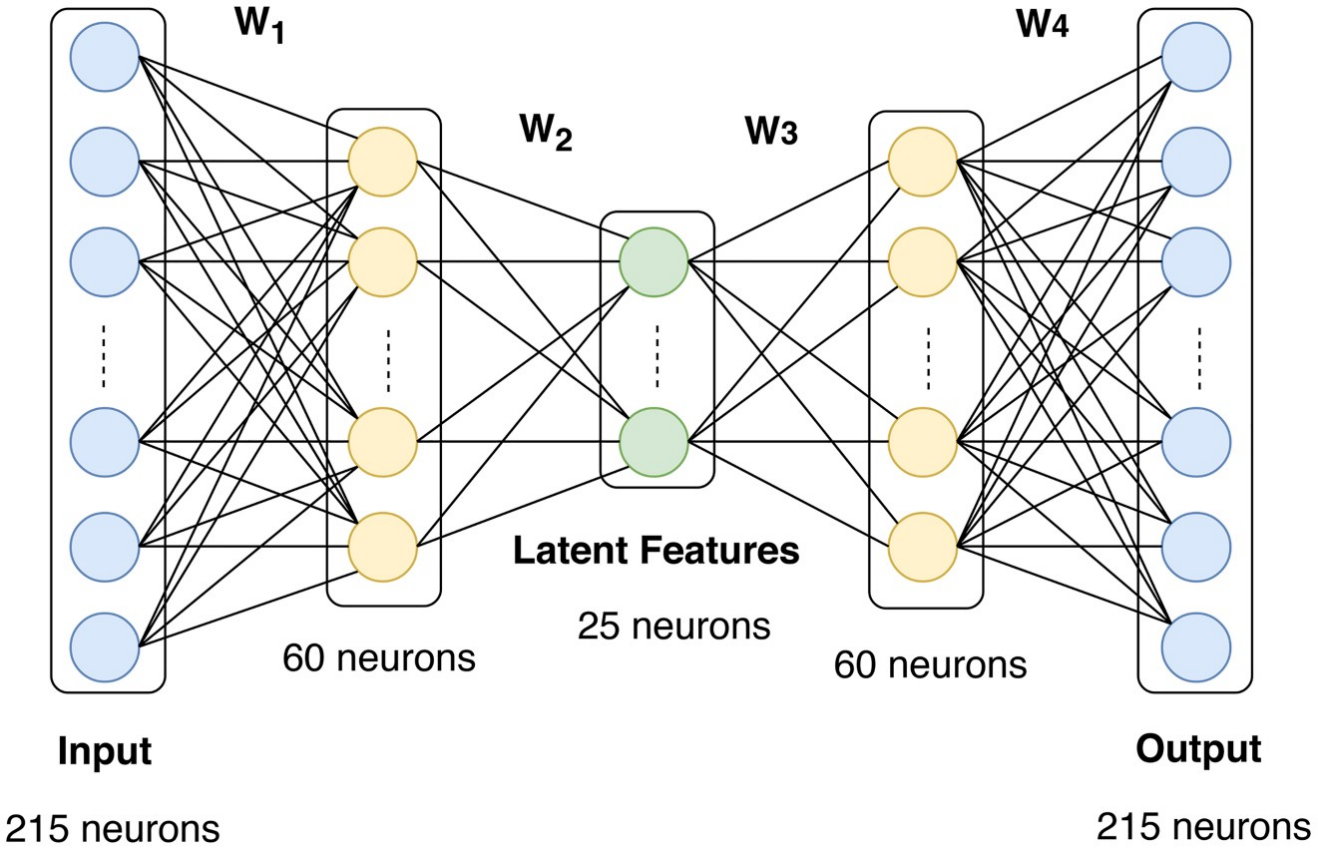
Architecture of the autoencoder used in the proposed model. It has three hidden layers with 60, 25, and 60 neurons in that order. The number of neurons for the output layer equals the number of raw features (215 in our case).

Autoencoders are neural network architectures that use unsupervised learning to extract compressed latent representations from unlabeled data. Fig 2 shows a schematic diagram of the autoencoder architecture that we used in our framework. It consists of one input layer, 3 hidden layers, and one output layer. The number of neurons in the input layer equals the number of raw features (215 in our case). The three hidden layers consist of 60, 25, and 60 neurons in that order. The number of neurons for the output layer equals the number of raw features (215 in our case). The output of layer *l* follows (Eq 6), where **x**_*l*_ is the input feature vector, **W**_*l*_ is edge weight matrix, and **b**_*l*_ is a bias term. We used the Rectified Linear Unit (ReLU(*x*) = max(0, *x*)) as the activation function since it has been shown to help the objective function converge faster [34]. The output of the autoencoder **x**′ is calculated as in Eq 7, where *m* denotes the number of layers of the autoencoder.

$$\begin{array}{c} f_{l}\left(x_{l}\right)=\text{ReLU}\left(W_{l}x_{l}+b_{l}\right) \end{array}$$ (6)

$$\begin{array}{c} x^{\prime }=F_{1\to m}\left(x\right)=f_{1}\circ \ldots .\circ f_{m}\left(x\right) \end{array}$$ (7)

The weights and biases of the autoencoder are learned by minimizing the error between the input **x** and the reconstructed input **x**′ (Eq 8), where *n* is the number of datapoints. In order to prevent over-fitting, an L2 regularization on the weights is added to the loss function with regularization parameter λ, where *m* denotes the number of layers (Eq 8). Additionally, in order to enforce the sparsity on the hidden layer neurons, we added Kullback-Leibler (KL) divergence [35] to the loss function (Eq 8), where *ρ* denotes the sparsity parameter, $\rho_{j}^{\prime }$ is the average activation, meaning output value, of neuron *j* in the latent representation layer of the autoencoder over all datapoints, *β* is a parameter that controls the weight of the sparsity penalty term, and *k* denotes the number of neurons on the latent representation layer. KL-divergence is a standard function to measure the difference between two distributions and by putting KL-divergence into the loss function, latent representation neurons are forced to activate a small fraction of their neurons [36]. This is useful to force the neurons to learn certain patterns of data which in turn increase their specificity in performance contrasted to the more general training. The sparse autoencoder is trained via the backpropagation algorithm [36] to minimize the loss function (*Loss*^(2)^). After the training is completed, the latent features are extracted and passed to the LSTM to train the model for phenotype prediction (food allergy vs. non-food allergy).

$$\begin{array}{c} {\text{Loss}}^{\left(2\right)}\left(x,x^{\prime }\right)=\frac{1}{n}\sum_{i=1}^{n}{\left\|x_{i}-x_{i}^{\prime }\right\|}^{2}+\lambda \sum_{j=1}^{m}{\left\|W_{j}\right\|}^{2}+\beta \sum_{j=1}^{k}\text{KL}(\rho ||\rho_{j}^{\prime }) \end{array}$$ (8)

## Experiments

### DIABIMMUNE dataset

**Fig 3:**
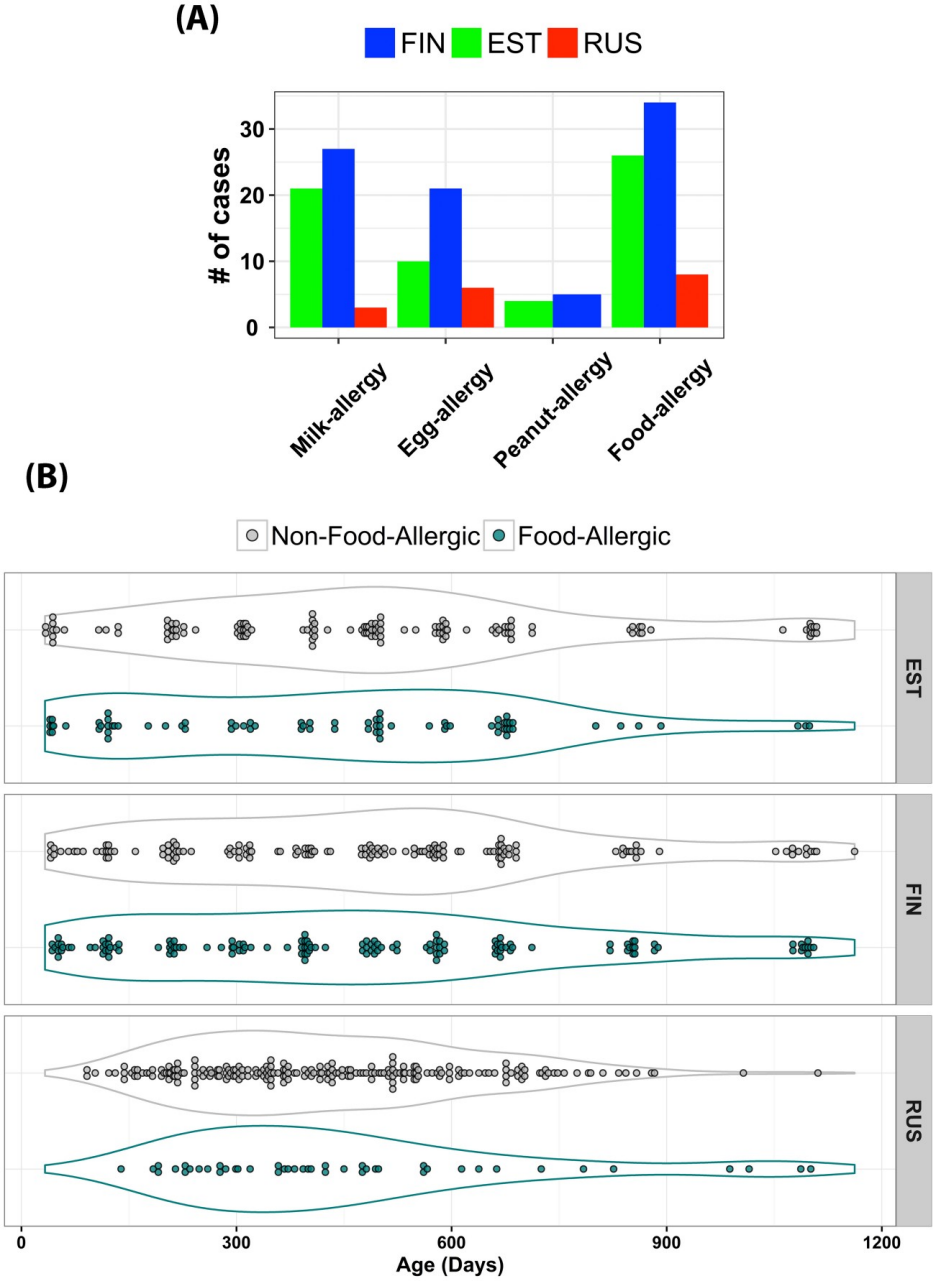
Summary of the DIABIMMUNE dataset. (A) Number of subjects allergic to milk, egg, and peanut within the DIABIMMUNE cohort after filtering out missing data. The food-allergy group is the summation of milk, egg, and peanut allergy. (B) Time distribution of 731 samples from 195 subjects (281 samples from 71 Finnish, 197 samples from 70 Estonian, and 253 samples from 54 Russian) stool samples sequenced using MGS from the DIABIMMUNE project. The collected samples have various forms of inconsistencies, such as different numbers of samples per subject.

In order to evaluate our proposed model, we used the longitudinal gut microbiome profiles from the DIABIMMUNE project (https://pubs.broadinstitute.org/diabimmune), a study that aimed to characterize host-microbe immune interactions contributing to autoimmunity and allergy. These diseases were evaluated in relationship to the hygiene hypothesis, which postulates that subjects with high bacterial exposure tend to have a more powerful immune system and fewer allergic diseases [12]. To test this hypothesis, stool samples were collected from 222 infants, equally distributed among three countries (74 from Russia, 74 from Finland, and 74 from Estonia) from birth to 3 years of age. At the time of stool sample collection, various food allergen-specific IgE levels were measured for each subject, and based on a predefined threshold, infants were annotated as allergic or non-allergic to the corresponding food allergen. Fig 3A shows the breakdown of the number of subjects with milk, egg, or peanut allergic responses. It is clear that the prevalence of the allergies is highest in Finland and lowest in Russia with Estonia intermediate, which is aligned with the hygiene hypothesis. For the purpose of evaluating our framework, we labeled subjects as food allergy positive if they are allergic to milk, eggs, or peanuts (Fig 3A).

As a preprocessing step, we removed all samples without a food allergy class label, i.e., missing data, resulting in 731 samples from 195 subjects (281 from 71 Finnish, 197 from 70 Estonian, and 253 from 54 Russian). The 195 subjects are categorized as 68 food allergic and 127 non-food allergic. Fig 3B shows the distribution of timepoints of samples collected from each class from each country. As shown, these samples suffer from all forms of variability, such as a different number of subjects per phenotypic group (food allergy vs non-food allergy), a different number of samples per subject, and samples not collected at consistent timepoints. Since our main objective in this study is to explore the capacity of LSTM to learn temporal representation of the microbial features, we removed samples from subjects with less than 3 timepoints. This results in keeping 658 samples from 148 subjects (256 from 56 Finnish, 238 from 43 Russian, and 164 from 49 Estonian), 52 subjects out of the 148 are food-allergic. These samples have been sequenced using MGS sequencing. As previously described in [12], reads were quality-controlled by filtering out low-quality reads, short reads (< 60 bp), and human reads. Taxonomic profiles were constructed using *MetaPhlAn2* [37]. The number of reads mapped to each taxonomic feature was then normalized to reads per kilobase per million (RPKM) of sample reads to correct for bias due to differences in genome size and sequencing depth. The aggregated taxonomic profiles of all 658 samples revealed 215 genera.

### Benchmarking procedure

We benchmarked the proposed LSTM model against other predictive models, such as Hidden Markov Model (HMM) [22], Multi-Layer Perceptron Neural Network (MLPNN) [38], Support Vector Machine (SVM) [39], Random Forests (RF) [40], and Least Absolute Shrinkage and Selection Operator (LASSO) [41]. In our evaluation, we benchmarked two aspects: (1) the effect on the prediction of using the latent representations versus features selected using traditional feature selection methods such as mRMR [33] or ranking based on variance, or using raw features, (2) the effect on the prediction of learning temporal dependency between the sequence of samples, as in LSTM or HMM, versus learning from each sample independently using methods such as MLPNN, SVM, RF, or LASSO. We used the mRMR method in the *praznik* R-package (https://cran.r-project.org/web/packages/praznik/index.html) to select top 25 microbial features that distinguish food-allergic samples from non-food-allergic samples. A major advantage of the mRMR method is the interpretability of the selected features. We also selected the top 25 most variable features.

We trained the previously described autoencoder and LSTM models separately using *Tensorflow* (v1.6.0) [42]. The autoencoder consists of 215, 60, 25, 60, and 215 neurons for the input layer, first hidden layer, second hidden layer (latent representation), third hidden layer, and output layer, respectively. We trained the model with the back-propagation algorithm using *Adam* optimizer [43] with a learning rate of 0.001 and a batch size of 5. The model was trained for 300 epochs, and the best model was saved based on the loss value on the test set. For L2 regularization we used λ = 0.05. For the sparsity constraint, we used *ρ* = 0.01 and *β* = 3. For the LSTM module, we used 64 neurons for the LSTM hidden neurons. Similar to the autoencoder, the LSTM model was trained with *Adam* Optimizer with a learning rate of 0.001 and batch size = 5. Similar to LSTM, MLPNN was implemented using *Tensorflow* (v1.6.0). Two hidden layers were used, the first has 128 neurons and the second has 256 neurons. Output layer has two neurons (number of classes). The network was trained with a learning rate of 0.001 for 50 epochs, and the best model was retrieved based on the validation set loss to be used on the test set. RELU was used as the activation function, and the network was regularized using dropout [44] with probability of dropping neurons (*p* = 0.5).

To benchmark our LSTM model against another model which can incorporate sequential data, we trained a hidden Markov model (HMM) using a Gaussian Mixture Model (GMM). We trained the HMM using the *hmmlearn* python library and trained in an unsupervised manner using two states and four mixtures over 100 iterations. The state labels were determined based on which labeling yielded the highest accuracy. Although GMM’s can be trained in a supervised fashion, they are usually used for clustering and are much more flexible than more classical clustering methods (e.g. k-means). The HMM models were first order and contained two states. The two methods are merged by forcing the emission probabilities of the HMM to follow a GMM. The RF, SVM, and LASSO models were all trained using Python’s *scikit-learn* package (http://scikit-learn.org). The RF models were trained by setting a maximum of 500 trees. All other parameters were left as the default values. The SVM models were trained using an exhaustive grid search with 5-fold cross-validation over the linear and Gaussian kernels, using the parameters 1, 10, 100, 1000 for error terms and the parameters 0.001, 0.0001 for *γ* values in Gaussian kernels. The LASSO models were trained using iterative fitting with 5-fold cross-validation for the error term *α* over a set of 50 numbers, evenly log-spaced between 4-10 and 0.5-10.

For the HMM model, the final state of the sequence was used for evaluation. In the case of RF, SVM, or LASSO, we trained the classifiers on the samples of the last timepoint of each subject because these methods do not have the capacity to learn temporal representation. This strategy ensures a fair comparison with LSTM, which uses all of a subject’s timepoints to predict the phenotype of the last timepoint.

### Evaluation metrics

To ensure robustness of the performance evaluation, we divided the data into test set (20%) and performed 10-fold cross-validation on the remaining 80% of the dataset. We repeated this process 10 times and each time we shuffled the dataset before dividing the data. Various performance metrics were calculated such as $Sensitivity=\frac{TP}{TP+FN}$ and $Specificity=\frac{TN}{TN+FP}$. These metrics have been used to obtain the area under the Receiver Operating Characteristic (auROC) curve. We also measured Matthew Correlation Coefficient ($MCC=\frac{TP*TN-FP*FN}{\sqrt{\left(TP+FP)(TP+FN)(TN+FP)(TN+FN\right)}}$) on the test set after each 10-fold. The MCC criterion is a way of evaluating classifiers considering all true positives (TP), false positives (FP), true negatives (TN) and false negatives (FN). A score of 1 indicates a perfect classification and a score of -1 indicates a completely incorrect classification.

Given the fact that our dataset is imbalanced (52 allergic and 96 non-allergic), we up-sampled samples from allergic infants and down-sampled samples from non-allergic infants. However, the change of the predictive power of the used classifiers was not significant (p-value<0.5, Mann-Whitney test), so we report here the results without up/down sampling procedures.

## Results and discussion

**Fig 4:**
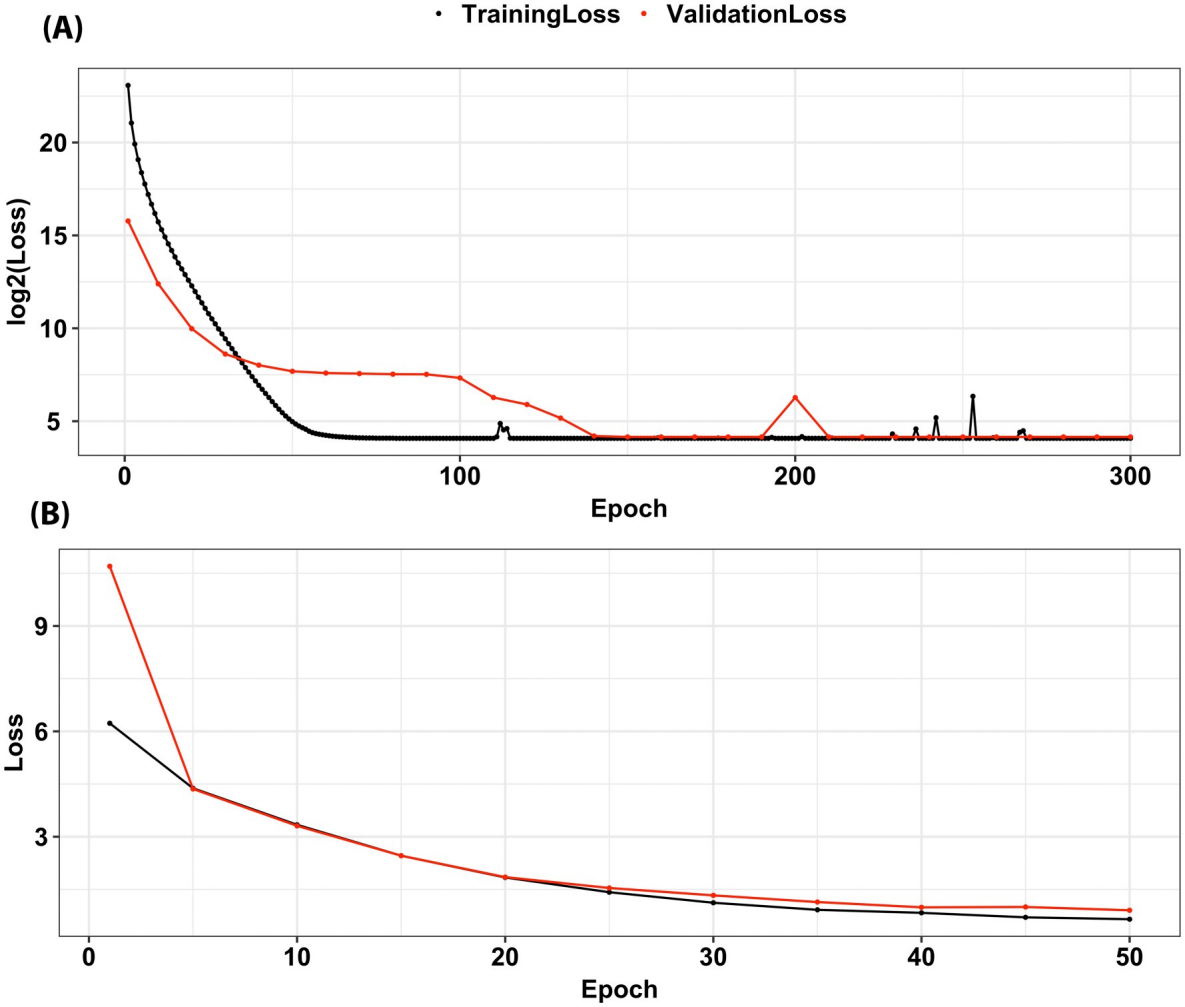
(A) Trajectory of training and validation loss function (*Loss*^(2)^) of the autoencoder with 60x25x60 architecture. The lowest loss on validation set was 16.79 and when the test set applied to this best model, the loss was 17.04. (B) Trajectory of training and validation loss function (*Loss*^(1)^) of the LSTM. The lowest loss on validation set was 0.91 and when the test set applied to this best model, the loss was 1.02

Firstly, we measured the loss function for several autoencoder architectures in order to select the most suitable architecture for our data (Table 1). For each architecture, the model with the lowest loss on the validation set was saved and then tested on the test set. Many of the tested architectures gave a similar loss on the validation set, however loss on test set varies. It tends to be small either when the autoencoder has many parameters (more neurons) or when the ratio between the number of neurons in the first layer to the latent layer is not high. Otherwise, loss on test set is high because either the latent layer does not have the capacity to learn a compressed representation (as in 50x25x50 or 50x15x50), or the first hidden layer does not learn enough compressed features to be passed to the latent layer (as in 100x25x100). Our criterion for architecture selection was that the autoencoder should yield small loss on test set while using as few parameters as possible. Giving this criterion, the autoencoder with 60x25x60 was our choice. Fig 4A shows the trajectory of the loss on the training set and validation set. The loss progressively decreases by more epochs until it stabilizes after 270 epochs. The loss never reaches zero due to the regularization we put in the autoencoder loss function to prevent over-fitting.

**Table 1:** Losses (training, validation, and testing) of the best trained model for several autoencoder architectures. The 60x25x60 architecture is the chosen model because it achieves small loss while using the least number of parameters.

Architecture	Training	Validation	Testing
150x50x150	16.95	17.26	16.90
150x25x150	16.90	18.13	16.59
150x15150	16.87	17.31	17.74
150x10x150	Model does not converge		
125x50x125	17.01	16.74	17.35
125x25x125	16.98	17.52	17.31
125x15x125	16.91	16.54	17.21
125x10x125	Model does not converge		
100x50x100	16.83	19.00	16.09
100x25x100	16.95	17.58	160.9
100x15x100	Model does not converge		
100x10x100	Model does not converge		
75x50x75	16.07	17.96	16.30
75x25x75	17.27	14.56	112.0
75x15x75	Model does not converge		
75x10x75	Model does not converge		
60x50x60	16.80	17.55	17.06
60x25x60	16.18	16.79	17.04
60x15x60	16.75	16.83	50.90
60x10x60	16.60	35.70	51.99
50x50x50	15.87	17.34	35.05
50x25x50	17.09	16.02	34.00
50x15x50	17.09	16.08	207.0
50x10x50	17.19	16.55	263.1

As highlighted previously, features selected using mRMR or variance-based methods have interpretability advantages over features extracted by the autoencoder. For example, the top selected genus by mRMR is *Faecalibacterium*, which is more abundant in the non-food allergy group, which is consistent with studies that associate *Faecalibacterium* with allergies, and specifically food-allergy [45, 46]. Other top selected features includes *Burkholderiales*, *Neisseria*, and *Enterococcus*. On the other hand, features with highest variability include: *Bifidobacterium*, *Bacteroides*, *Prevotella*, *Escherichia*, *Klebsiella*, and *Faecalibacterium*.

Subsequently, we evaluated how our proposed LSTM model compares with the commonly used classification methods. Table 2 summarizes the evaluation of auROC and MCC on the test set using 10 times 10-fold cross-validation experiments. In this table, we evaluated six classifiers; LSTM, HMM, MLPNN, RF, SVM, and LASSO. For each classifier, we evaluated four types of input features; latent features which extracted from the trained autoencoder, 25 features selected by mRMR method, 25 most variable features, and 215 raw taxonomic profile features. P-values are calculated using Mann-Whitney U test between LSTM-mRMR-25 versus each corresponding model since LSTM-mRMR-25 achieved the highest auROC and MCC among all benchmarked models.

**Table 2:** Evaluation of auROC and MCC for the proposed LSTM model versus baseline models. In this table, we evaluated six classifiers; LSTM, HMM, MLPNN, RF, SVM, and LASSO. For each classifier, we evaluated four types of input features; latent features which extracted from the trained autoencoder, 25 features selected by mRMR method, 25 most variable features, and 215 raw taxonomic profile features. The auROC and MCC results shown below are the average of auROC and MCC measured on the test set. The experiments were repeated 10 times and samples were shuffled after each 10-fold cross-validation to test the robustness of each classifier. P-values were calculated using Mann-Whitney U test between LSTM-mRMR-25 versus each corresponding method.

Model	Features	auROC [mean (sd)]	auROC [p-value]	MCC [mean (sd)]	MCC [p-value]
**LSTM**	**latent-25**	0.67 (0.07)	4.20E-01	0.29 (0.22)	3.47E-03
	**mRMR-25**	0.69 (0.09)	1	0.40 (0.19)	1
	**var-25**	0.61 (0.12)	7.00E-02	0.23 (0.20)	9.51E-04
	**raw**	0.65 (0.10)	1.70E-01	0.37 (0.18)	5.10E-02
**HMM**	**latent-25**	0.52 (0.06)	5.99E-22	0.14 (0.20)	2.25E-12
	**mRMR-25**	0.55 (0.08)	8.92E-16	0.21 (0.19)	3.67E-07
	**var-25**	0.52 (0.07)	4.68E-22	0.16 (0.15)	1.18E-10
	**raw**	0.50 (0.07)	5.63E-25	0.06 (0.21)	7.66E-17
**MLPNN**	**latent-25**	0.54 (0.08)	5.50E-04	0.11 (0.13)	5.93E-06
	**mRMR-25**	0.57 (0.06)	1.95E-03	0.18 (0.11)	5.95E-03
	**var-25**	0.54 (0.03)	6.66E-05	0.05 (0.07)	1.13E-05
	**raw**	0.63 (0.05)	4.93E-02	0.12 (0.12)	1.13E-03
**RF**	**latent-25**	0.44 (0.14)	3.00E-22	0.05 (0.25)	1.99E-15
	**mRMR-25**	0.63 (0.13)	6.73E-02	0.23 (0.23)	6.00E-03
	**var-25**	0.57 (0.12)	1.08E-07	0.08 (0.23)	2.31E-15
	**raw**	0.59 (0.13)	2.00E-03	0.14 (0.23)	2.71E-11
**SVM**	**latent-25**	0.52 (0.15)	6.49E-12	0.06 (0.21)	9.20E-31
	**mRMR-25**	0.57 (0.13)	1.46E-07	-0.04 (0.23)	1.64E-28
	**var-25**	0.46 (0.15)	1.45E-20	0.03 (0.22)	9.20E-31
	**raw**	0.52 (0.14)	7.21E-13	-0.04 (0.23)	1.23E-27
**LASSO**	**latent-25**	0.51 (0.14)	1.51E-14	-0.04 (0.21)	5.90E-27
	**mRMR-25**	0.59 (0.12)	2.00E-04	0.01 (0.23)	1.45E-26
	**var-25**	0.59 (0.13)	3.20E-05	-0.17 (0.01)	2.36E-30
	**raw**	0.57 (0.14)	1.00E-03	-0.17 (0.00)	1.66E-30

In general, LSTM outperformed the other classifiers, supporting the concept that learning a sequence of events increases the prediction power. While HMM has the capability to learn time sequences, it fails to work on the current dataset mainly because the DIABIMMUNE, similar to all human longitudinal studies, suffers from sampling inconsistency, while sampling consistency is required by the HMM to learn enough information. In general, the variability in auROC of LSTM and HMM is smaller compared to methods that do not learn time dependency. LSTM trained with the top 25 features selected by mRMR gave the best performance measured by auROC and MCC. Generally, latent features learned by autoencoder are not associated with high performance in all tested models. One reason for the low performance of the latent representation is that the way they have learned to extract the compressed representation from microbial profiles was without considering the phenotype in the loss function. All other classifiers that do not consider time sequence data perform poorly compared to LSTM, supporting the concept that learning a sequence of events increases the prediction power. Additionally, we have investigated the usage of 50 latent features, 50 features selected by mRMR, and top 50 features ranked by variance, but they all show a decrease in prdictive performance compared to the 25 features selected by each of the aforementioned methods.

### Execution time

The execution time of training LSTM on different types of features is comparable and depends on the number of epochs and batch size. 10-times 10-fold cross validation took, on average, 92 minutes on the DIABIMMUNE dataset given all the parameters stated above. On the other hand, on average, HMM took 9 minutes, MLPNN took 33 minutes, SVM took 12.5 minutes, RF took 7 minutes, and LASSO took 1.5 minutes. The prediction time is linear with respect to the size of the test set. The evaluation was conducted on a MAC machine with 2.5 GHz Intel Core i7 processor and 16 GB 1600 MHz RAM. No GPU was used for training of the LSTM.

## Conclusion

Food allergy is usually difficult to diagnose at young ages, and the inability to diagnose patients with this atopic disease at an earlier age may lead to severe complications due to the lack of treatment. In this work, we have developed a deep learning framework that has the capacity to predict food allergy from longitudinal gut microbiome profiles. The framework is based on Long Short-Term Memory (LSTM) networks with a feature selection procedure. To effectively choose features prior to LSTM training, we have evaluated four procedures, including sparse autoencoder for extracting potential latent representation in microbiome, Minimum Redundancy Maximum Relevance, ranking based on variance, and raw taxonomic profile features. We tested the framework on the DIABIMMUNE dataset, a study that aimed to characterize host-microbe interactions contributing to autoimmunity and allergy. Our results demonstrate the increase in predictive power of the LSTM model compared to Hidden Markov Model, Multi-Layer Perceptron Neural Network, Random Forest, SVM, and LASSO regression. The LSTM models coupled with the mRMR selected features achieved the best performance in our evaluation.

Although our deep learning framework shows the potential to predict allergic phenotype from a sequence of gut microbiome profiles and outperforms other classical methods, it does not reach a prediction level for optimal clinical utilization. This is mainly due to the nature of the training dataset that we used to train our model. The DIABIMMUNE dataset is small and each subject has a few timepoints (6 on average). With the further reduction in sequencing costs, we anticipate that data from multiple large-scale longitudinal microbiome projects will be available which in turn could be used to train models like ours for better prediction power. As more data becomes available, there is also the potential to explore transfer learning, where the information found in models trained on one task is used to improve the prediction of models trained on another task. This has the potential of allowing longitudinal microbiome profiling that can be used for predicting diseases before they are clinically apparent, especially for autoimmune diseases that are linked to host microbiome interactions, such as asthma and diabetes mellitus. The project source code is publicly available on (https://github.com/aametwally/FoodAllergyPredictor).
